# Neuropilin-1 Associated Molecules in the Blood Distinguish Poor Prognosis Breast Cancer: A Cross-Sectional Study

**DOI:** 10.1038/s41598-017-03280-0

**Published:** 2017-06-12

**Authors:** Adviti Naik, Noura Al-Zeheimi, Charles Saki Bakheit, Marwa Al Riyami, Adil Al Jarrah, Mansour S. Al Moundhri, Zamzam Al Habsi, Maysoon Basheer, Sirin A. Adham

**Affiliations:** 1 Department of Biology, College of Science, Sultan Qaboos University, P. O. Box 36, 123 Muscat, Oman; 2 Department of Mathematics and Statistics, Sultan Qaboos University, P. O. Box 36, 123 Muscat, Oman; 3 Department of Pathology, College of Medicine, Sultan Qaboos University, P.O.Box 35, 123 Muscat, Oman; 4 Department of Surgery, Sultan Qaboos University Hospital, P. O. Box 35, 123 Muscat, Oman; 5 Department of Medicine, College of Medicine, Sultan Qaboos University, P. O. Box 35, Muscat, Oman

## Abstract

Circulating plasma and peripheral blood mononuclear (PBMCs) cells provide an informative snapshot of the systemic physiological state. Moreover, they provide a non-invasively accessible compartment to identify biomarkers for personalized medicine in advanced breast cancer. The role of Neuropilin-1 (NRP-1) and its interacting molecules in breast tumor tissue was correlated with cancer progression; however, the clinical impact of their systemic levels was not extensively evaluated. In this cross-sectional study, we found that circulating and tumor tissue expression of NRP-1 and circulating placental growth factor (PlGF) increase in advanced nodal and metastatic breast cancer compared with locally advanced disease. Tumor tissue expression of NRP-1 and PlGF is also upregulated in triple negative breast cancer (TNBC) compared to other subtypes. Conversely, in PBMCs, *NRP-1* and its interacting molecules *SEMA4A* and *SNAI1* are significantly downregulated in breast cancer patients compared to healthy controls, indicating a protective role. Moreover, we report differential PBMC expression profiles that correlate inversely with disease stage (*SEMA4A, SNAI1, PLXNA1* and *VEGFR3*) and can differentiate between the TNBC and non-TNBC tumor subtypes (*VEGFR3* and *PLXNA1*). This work supports the importance of NRP-1-associated molecules in circulation to characterize poor prognosis breast cancer and emphasizes on their role as favorable drug targets.

## Introduction

Breast cancer is the second most common cause of cancer-related deaths in females, with an estimated 41,000 deaths in the USA in 2016. Although, the 5-year relative survival rate has increased to approximately 90% in the past decade^1^. Extensive research in breast cancer management has led to the development of cytotoxic treatments and biology-driven targeted therapeutics contributing to improved survival^2^. Despite the improvements in breast cancer management there still remain challenges in diagnosing and treating poor prognosis types of breast cancer, combating tumor heterogeneity. Hence, the validation of appropriate biomarkers to fine tune the choice of treatment in a personalized fashion is of a high priority.

Comprehensive research has been conducted on cancer cells and tissues in comparison to circulating molecules and cells in the plasma, which can be easily sampled, have an extraordinary dynamic range, broad heterogeneity and tremendous diagnostic and therapeutic prospects, and thus its potential still remains untapped^3^. The plasma proteome, which is increasingly being elucidated, and peripheral blood mononuclear cells (PBMCs) provide a snapshot of the molecular, physiological and pathological characteristics of the living system. PBMCs, comprising of T cells, B cells, monocytes and natural killer cells (NK), provide the primary response to defend against malignancy and hence, deregulations in these cells as a result of tumor immune evasion can be determined at early disease stages^4^. Several studies investigating differential expression in PBMCs in pancreatic cancer^5^, non-small cell lung cancer^6^ and renal cancer^7, 8^ revealed unique expression profiles of diagnostic/prognostic value.

Neuropilins are non-tyrosine kinase receptors with a multitude of functions dependent on the binding of specific ligands. Accumulating evidence indicates the role of Neuropilin-1 (NRP-1) in promoting cancer by binding to molecules involved in angiogenesis and the Epithelial-to-Mesenchymal Transition (EMT) pathway in tumor tissue such as vascular endothelial growth factor (VEGF)^9^, and its receptors^10^, placental growth factor (PlGF)^9^, and transforming growth factor beta 1 (TGFβ1)^11^. Apart from the full-length isoform of NRP-1, it is also present as soluble isoforms generated by alternative splicing^12^. Blocking NRP-1 with an anti-NRP-1 antibody increased the circulating levels of the soluble PlGF, VEGF and NRP-1 in advanced solid tumors in a phase I clinical trial^13^. An alternative VEGF receptor involved in lymphangiogenesis, VEGFR3, was shown to interact with the VEGFR2/NRP-1 complex on VEGF-C binding to activate AKT signaling^14^. NRP-1, on forming a complex with plexins also interacts with the secreted class 3 semaphorins (SEMA3 A,B,C,D,F) that are antagonistic to VEGF signaling, hence promoting tumor apoptosis and inhibiting cell migration^15^. SEMA4A binding to NRP-1 has been established in T regulatory (Treg) immune cells, wherein it stabilizes the intra-tumoral Treg cells and enhances tumor immune evasion^16^. Additionally, numerous transcriptional regulators act downstream of NRP-1 such as SNAI1^17^, a transcriptional repressor pivotal for cell differentiation and survival that reverses the tumor suppressive effects induced by TGFβ1 in early stage breast cancer^18^. In a previous study on ovarian cancer we showed that NRP-1 had a positive relation to EMT markers^19^.

NRP-1 plays a functional role in the immune system^20^, where it is involved in primary immune response initiation by mediating the interaction between dendritic cells and resting T cells^21^. Moreover, at intra-tumoral sites NRP-1 plays a role in evasion of immune surveillance using various mechanisms^16^. Extrapolating from these observations, we hypothesized that the altered expression of *NRP-1*, its associated growth factors and commonly known partner molecules in PBMCs as well as plasma of breast cancer patients can serve as a good target to identify a potential biomarker for more personalized diagnostic and treatment plans. Our work illustrates that the upregulated levels of circulating NRP-1 and PlGF associate with nodal and distant metastasis in breast cancer. The expression of NRP-1 in the breast tumor tissue displays a similar pattern to its plasma counterpart. Additionally, the tissue expression of both NRP-1 and PlGF was significantly higher in the tissues of triple negative breast cancer (TNBC) cases compared to the other molecular subtypes. Moreover, we have identified distinct PBMC expression profiles that correlate with poor prognosis types of breast cancer. We highlight cancer-specific down regulation of *NRP-1*, *SNAI1* and *SEMA4A* in PBMCs with a further decrease of *SNAI1, SEMA4A, VEGFR3* and *PLXNA1* in patients with advanced disease. We demonstrate the ability of *VEGFR3* and *PLXNA1* expression in PBMCs to distinguish triple negative breast cancer (TNBC) from other subtypes. We finally accentuate age-dependent differential trends in gene expression between early onset breast cancer and older patients, which have critical implications for the choice of treatment.

## Results

### Patient’s cohort characteristics

Seventy female breast cancer patients were included in the study, with a median age of 45 years and a range between 19–68 years; about 75% of the cohort was above 35 years. All patients’ disease was characterized and staged according to the American Joint Committee on Cancer (AJCC) standards in a multidisciplinary team meeting consisting of surgeons, pathologists, oncologists, radiologists, geneticists and gynecologists. The most common molecular types of breast cancer in the cohort are luminal B (28.6%), Luminal B-like (27.1%), Her-2 type (18.6%) and triple negative (11.4%) with the lowest frequency of the luminal A type (7.1%). The majority of the cases were grade 3 (47.1%) and grade 2 (35.7%) with the proliferation index ranging from 14–80% (74.4%). In addition, 11.4% of the cohort studied was diagnosed with distant metastases. Moreover, a large proportion of the cohort had nodal metastasis (68.5%) and large tumor sizes greater than 2 cm (81.4%) (Details of tumor size is in methods section) (Table 1).

**Table 1:** Clinical and pathological characteristics of breast cancer patients.

CATEGORY	SUBTYPE	CASE PERCENTAGE (CASE FREQUENCY)
Age group	18–35 years	24.3% (17/70)
	>35 years	75.5% (53/70)
Tumor Molecular type	Luminal B	28.6% (20/70)
	Luminal B-like	27.1% (19/70)
	Luminal A	7.1% (5/70)
	Her-2 type	18.6% (13/70)
	Triple Negative	11.4% (8/70)
	Unknown	7.1% (5/70)
Ki-67 Proliferation Index	<14%	8.5% (6/70)
	14–30%	28.6% (20/70)
	31–50%	22.9% (16/70)
	51–80%	22.9% (16/70)
	>80%	10% (7/70)
	Unknown	7.1% (5/70)
Tumor Grade	Grade 1	8.5% (6/70)
	Grade 2	35.7% (25/70)
	Grade 3	47.1% (33/70)
	Unknown	8.5% (6/70)
Node Status	N0	25.7% (18/70)
	N1	47.1% (33/70)
	N2	12.9% (9/70)
	N3	8.5% (6/70)
	Unknown	5.7% (4/70)
Tumor size	T1 (<2 cm)	11.4% (8/70)
	T2	41.4% (29/70)
	T3 (>5 cm)	15.7% (11/70)
	T4 (tumors breaching the chest wall/skin or inflammatory breast cancer	24.3% (17/70)
	Unknown	7.1% (5/70)
Metastasis Status	M0	75.7% (53/70)
	M1	11.4% (8/70)
	Unknown	7.7% (9/70)
Overall stage	Stage 0	2.9% (2/70)
	Stage 1	8.6% (6/70)
	Stage 2	38.6% (27/70)
	Stage 3	34.3% (24/70)
	Stage 4	11.4% (8/70)
	Unknown	4.3% (3/70)

### Circulating NRP-1 increases with nodal/distant metastasis and large tumor size

**Figure 1:**
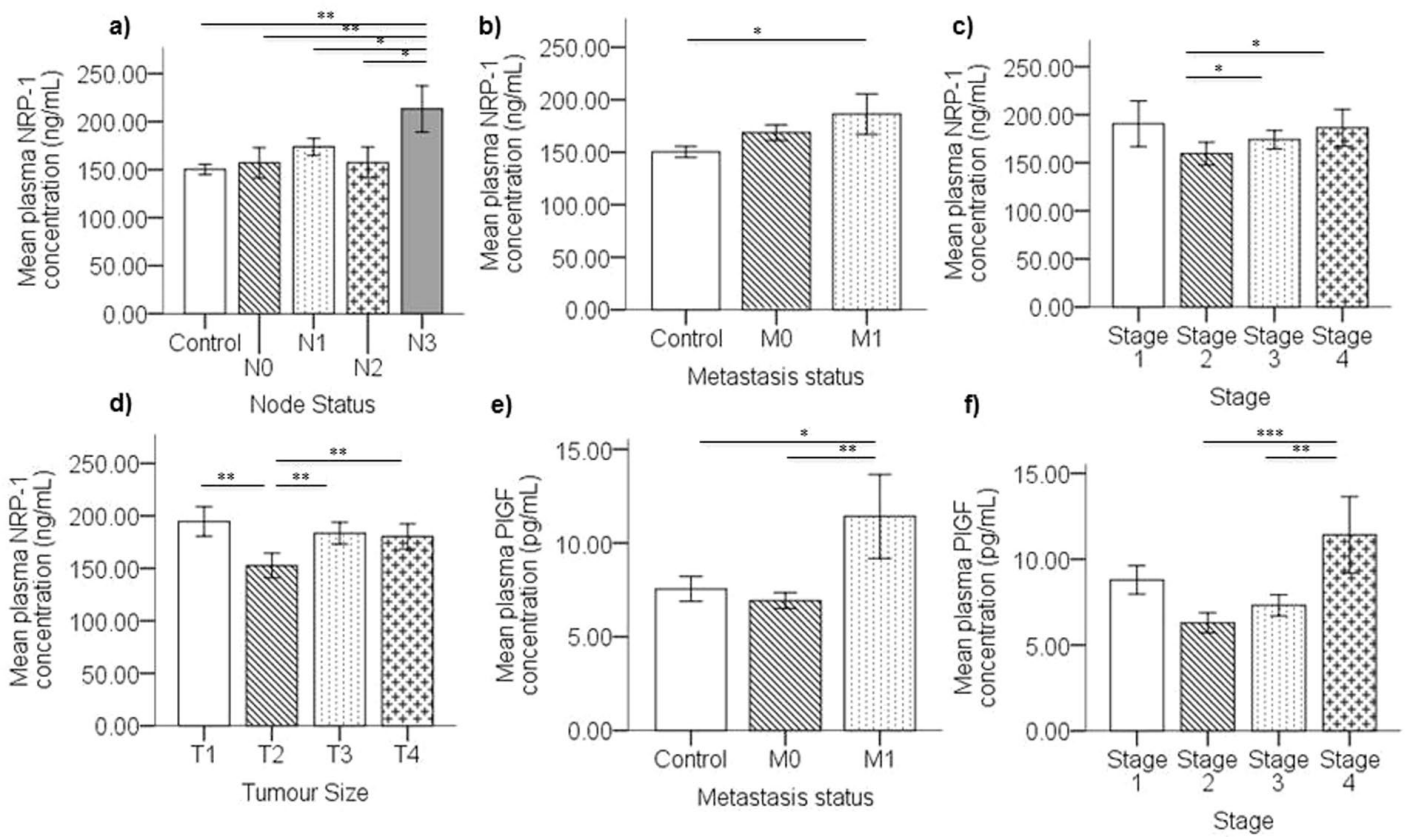
Elevated plasma NRP-1 and PlGF associates with advanced breast cancer. The graphs represent mean concentration of plasma NRP-1 ± SEM measured by ELISA in breast cancer patients which indicates significant upregulation in (**a**) advanced nodal disease and (**b**) metastasis, (**c**) advanced disease stage and (**d**) tumor size. (**e**) Plasma PlGF mean concentration ± SEM shows significantly upregulated levels in metastatic breast cancer cases compared to non-metastatic and healthy controls and (**f**) upregulation in Stage 4 cases compared to stage 2 and 3. p < 0.05 considered to indicate statistical significance. *p < 0.05, **p < 0.01, ***p < 0.001, Multivariate ANOVA followed by Fisher’s LSD post hoc test.

Plasma levels of both NRP-1 and PlGF were quantified in healthy controls and breast cancer patients and correlated to patient disease characteristics. Multivariate analysis, with plasma NRP-1 and PlGF as dependent variables (DV), indicated that circulating NRP-1 was significantly altered according to the patients’ nodal status (F_(4,97)_ = 3.309, *p* = *0.014*). Breast cancer patients with advanced diseased nodes N3 displayed significantly higher plasma NRP-1 compared to patients with no nodal metastasis N0 (*p* = *0.003*), less advanced nodal metastasis N1 (*p* = *0.023*) and N2 (*p* = *0.018*) and to healthy controls (*p* = *0.002*) (Fig. 1a). This result was confirmed by univariate analysis (Table 2). Plasma NRP-1 also significantly varied with the patient metastasis status (F_(2,99)_ = 5.481, *p* = *0.006*). Metastatic breast cancer patients displayed upregulated plasma NRP-1 compared to healthy controls (*p* = *0.028*) (Fig. 1b), although univariate analysis also indicated increased plasma NRP-1 in non-metastatic breast cancer patients compared to healthy controls (Table 2). As expected from the nodal and metastasis status observations, plasma NRP-1 was also significantly altered according to the overall disease stage (F_(3,47)_ = 4.914, *p* = *0.001*). Stage 3 (*p* = *0.032*) and Stage 4 (*p* = *0.025*) cases displayed significantly upregulated circulating NRP-1 compared to Stage 2 cases, although the levels at Stage 1 appears similar to Stage 4 (Fig. 1c). In addition, cases with tumor size T1 (*p* = *0.002*), T3 (*p* = *0.003*) and T4 (*p* = *0.002*) also displayed increased level of circulating NRP-1 compared to T2 tumor size cases (F_(3,48)_ = 6.592, *p* = *0.001*) (Fig. 1d).

**Table 2:** Univariate analysis for multiple comparisons.

Characteristic	Protein/Gene			Mean diff	P value
Node status	Plasma NRP-1	N3	N0	56.1210	**0.020**
			N2	55.8930	**0.030**
			Controls	62.8562	**0.004**
	Tissue NRP-1	N3	N0	1.9631	**0.041**
			N1	2.1078	**0.023**
Metastasis status	Plasma NRP-1	M0	Controls	18.3318	**0.048**
	Plasma PlGF	M1	Controls	36.0282	**0.042**
		M1	M0	4.4868	**0.006**
			Controls	3.8616	**0.016**
	Tissue NRP-1	M1	M0	1.92737	**0.016**
Tumor Molecular Type	Tissue NRP-1	TNBC	Lum B	1.9856	**0.043**
			Lum B-like	2.8738	**0.005**
	Tissue PlGF	TNBC	Lum B	1.5988	**0.026**
Tumor Size	PBMC *SNAI1*	T1	T3	0.9656	**0.019**
	PBMC *SEMA4A*	T1	T3	0.6774	**0.014**
			T4	0.5302	**0.035**
	PBMC *VEGFR3*	T1	T3	0.8202	**0.018**
			T4	0.8965	**0.005**
	PBMC *PLXNA1*	T1	T4	0.25418	**0.048**
Stage	Plasma PlGF	Stage 4	Stage 2	5.1223	**0.001**
			Stage 3	4.1061	**0.007**
	Tissue NRP-1	Stage 4	Stage 3	2.0989	**0.012**
	PBMC *SNAI1*	Stage 1	Stage 2	0.9699	**0.014**
			Stage 3	0.8934	**0.025**
			Stage 4	0.9314	**0.045**
	*PBMC SEMA4A*	Stage 1	Stage 3	0.5479	**0.034**
			Stage 4	0.6942	**0.022**
	*PBMC VEGFR3*	Stage 1	Stage 4	0.8795	**0.027**
		Stage 2	Stage 4	0.5879	**0.049**
	*PBMC PLXNA1*	Stage 1	Stage 4	1.0125	**0.001**
		Stage 2	Stage 4	0.6087	**0.009**
		Stage 3	Stage 4	0.6774	**0.005**

### Plasma PlGF Associates with Distant Metastasis

Similar to plasma NRP-1, multivariate analysis indicated that the levels of plasma PlGF are significantly altered with distant metastasis (F_(2,99)_ = 5.481, *p* = *0.006*), however no significant association was found with nodal metastasis. Increased circulating PlGF was observed in metastatic patients compared to patients with no distant metastasis (*p* = *0.006*) and healthy controls (*p* = *0.016*) (Fig. 1e). The result was also confirmed by univariate analysis (Table 2). Additionally, patients diagnosed with Stage 4 disease had significantly higher PlGF levels compared to Stage 2 (*p* = *0.001*) and Stage 3 cases (*p* = *0.007*) and an increased trend compared to Stage 1 (Fig. 1f) (Table 2).

### Upregulated tissue NRP-1 and PlGF in poor prognosis breast cancer

**Figure 2:**
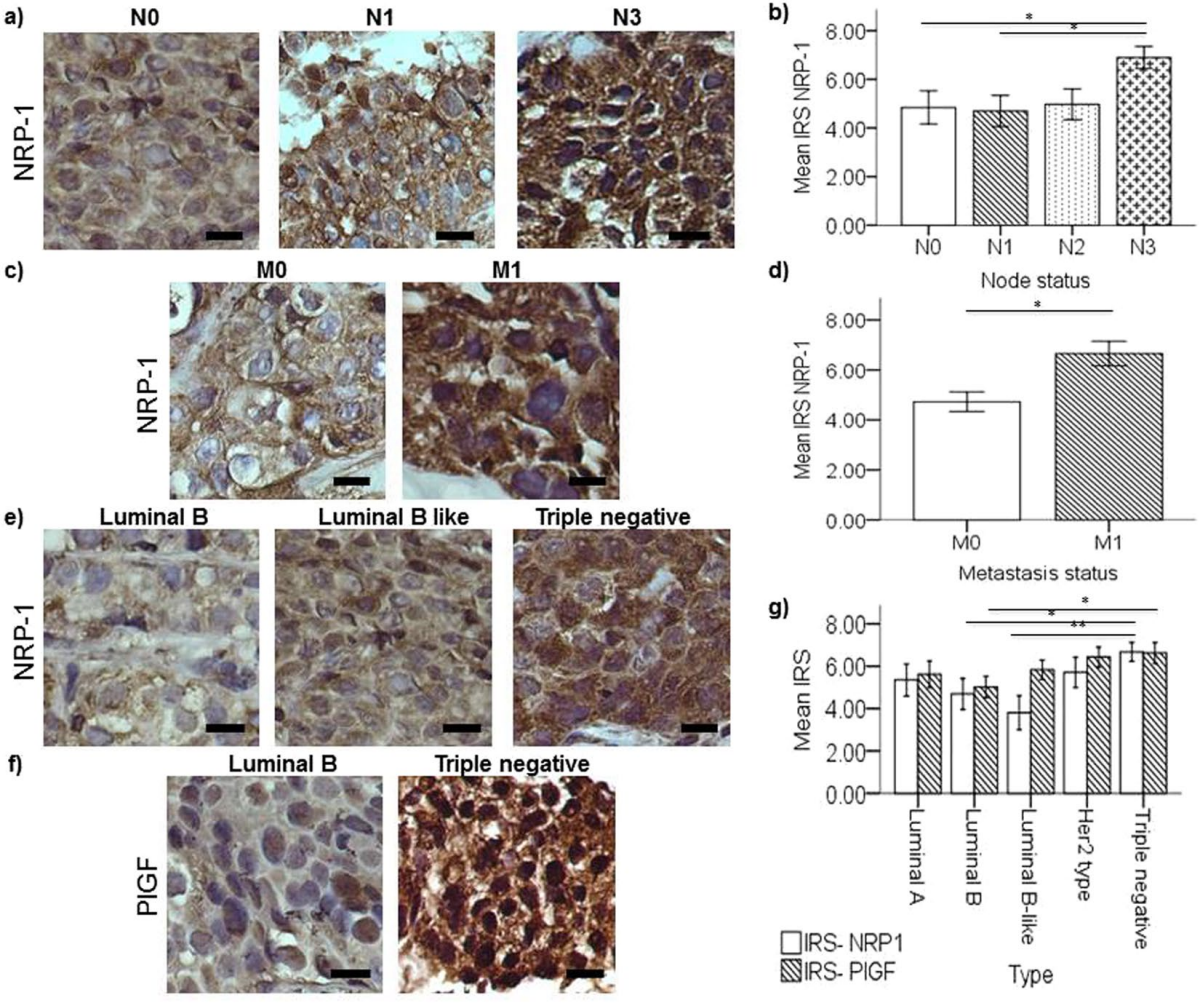
NRP-1 and PlGF tumor tissue expression associated with poor prognosis breast cancer. Representative immunohistochemical staining images for NRP-1 tumor tissue expression according to patient subgroups based on (**a**,**b**) nodal status, (**c**,**d**) metastatic status; and (**e–g**) NRP-1 and PlGF tumor expression in breast cancer patients tissues grouped according to their tumor molecular subtype. Graphs represent calculated mean immuno-reactive score (IRS) of NRP-1 and/or PlGF expression ± SEM in breast tumor tissue grouped according to the respective subtypes. NRP-1 tumor tissue expression was significantly higher in cases with metastasis and advanced nodal disease. Moreover, NRP-1 was significantly upregulated in TNBC cases compared to luminal B and luminal B-like; however, PlGF was significantly higher in TNBC cases compared to luminal B subtype only. p < 0.05 considered to indicate statistical significance. Scale bar = 50 µm, *p < 0.05, **p < 0.01, Multivariate ANOVA followed by Fisher’s LSD post hoc test.

In order to determine the association between plasma NRP-1 and PlGF and their corresponding expression in the tumor tissue, immunohistochemical staining was carried out on breast tumor tissue representative of the nodal and metastatic status subgroups. Pearson’s correlation test indicated a low but significant positive correlation between the tissue expression of NRP-1 and PlGF (Pearson’s correlation = 0.364, *p* = *0.002*). Similar to observations in the plasma, breast cancer cases with advanced nodal metastasis N3 (n = 14) have higher tumor NRP-1 tissue expression compared to cases with no nodal metastasis (n = 18, *p* = *0.041*) or N1 (n = 22, *p* = *0.023*) disease, as indicated by both multivariate and univariate analysis (Fig. 2a and b, Table 2). Tumor tissue expression of NRP-1, but not PlGF, was significantly upregulated in metastatic breast cancer (n = 15) compared to non-metastatic cases (n = 52, *p* = *0.016*) (Fig. 2c and 2d, Table 2). Extrapolating from these observations, NRP-1 tumor tissue expression was also upregulated in Stage 4 (n = 15) compared to Stage 3 (n = 38, *p* = *0.012*) breast cancer cases (Supplementary Fig. S1, Table 2). Additionally, TNBC cases (n = 14) had significantly increased NRP-1 expression in tumor tissue compared to the luminal B (n = 16, *p* = *0.043*) and B-like (n = 14, *p* = *0.005*) subtypes and higher PlGF expression compared to luminal B cases (n = 16, *p* = *0.026*) (Fig. 2e–g, Table 2).

### *NRP-1, SNAI1* and *SEMA4A* expression decreases in PBMCs in breast cancer

**Figure 3:**
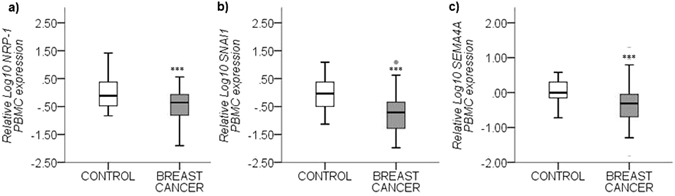
Differential PBMC gene expression between healthy controls vs breast cancer patients. Graphs **(a**–**c)** represent the mean relative log_10_ transformed gene expression (±SEM) as quantified using quantitative real time qRT-PCR. The relative expression of the following genes is significantly downregulated in PBMCs isolated from breast cancer patients compared to healthy controls: (**a**) Log_10_ *NRP-1*, (**b**) Log_10_ *SNAI1* and (**c**) Log_10_ *SEMA4A*. Target gene expression levels are normalized against *GUSB* gene expression and scaled to expression levels in control cases. P < 0.05 considered to indicate statistical significance. ***p < 0.001, Independent samples T-test.

In addition to plasma and tissue assessment of NRP-1 and PlGF, the expression of *NRP-1* and its key ligands were assessed in patients’ PBMCs and compared to healthy controls. As depicted in Fig. 3a–c, breast cancer patients displayed significantly decreased expression of *NRP-1* (*p* < *0.0001*), *SNAI1* (*p* < *0.0001*) and *SEMA4A (p* < *0.0001*) in PBMCS compared to healthy controls. However, no significant differences were observed for any of the other genes tested between controls and breast cancer patients. The expression *of NCAD, SLUG, VEGFR1, VEGFR2* and *SEMA3A* could not be detected using quantitative real-time PCR in neither control nor patient PBMCs.

### *SNAI1*, *SEMA4A*, *VEGFR3* and *PLXNA1* in PBMCs inversely relates with large tumor size

**Figure 4:**
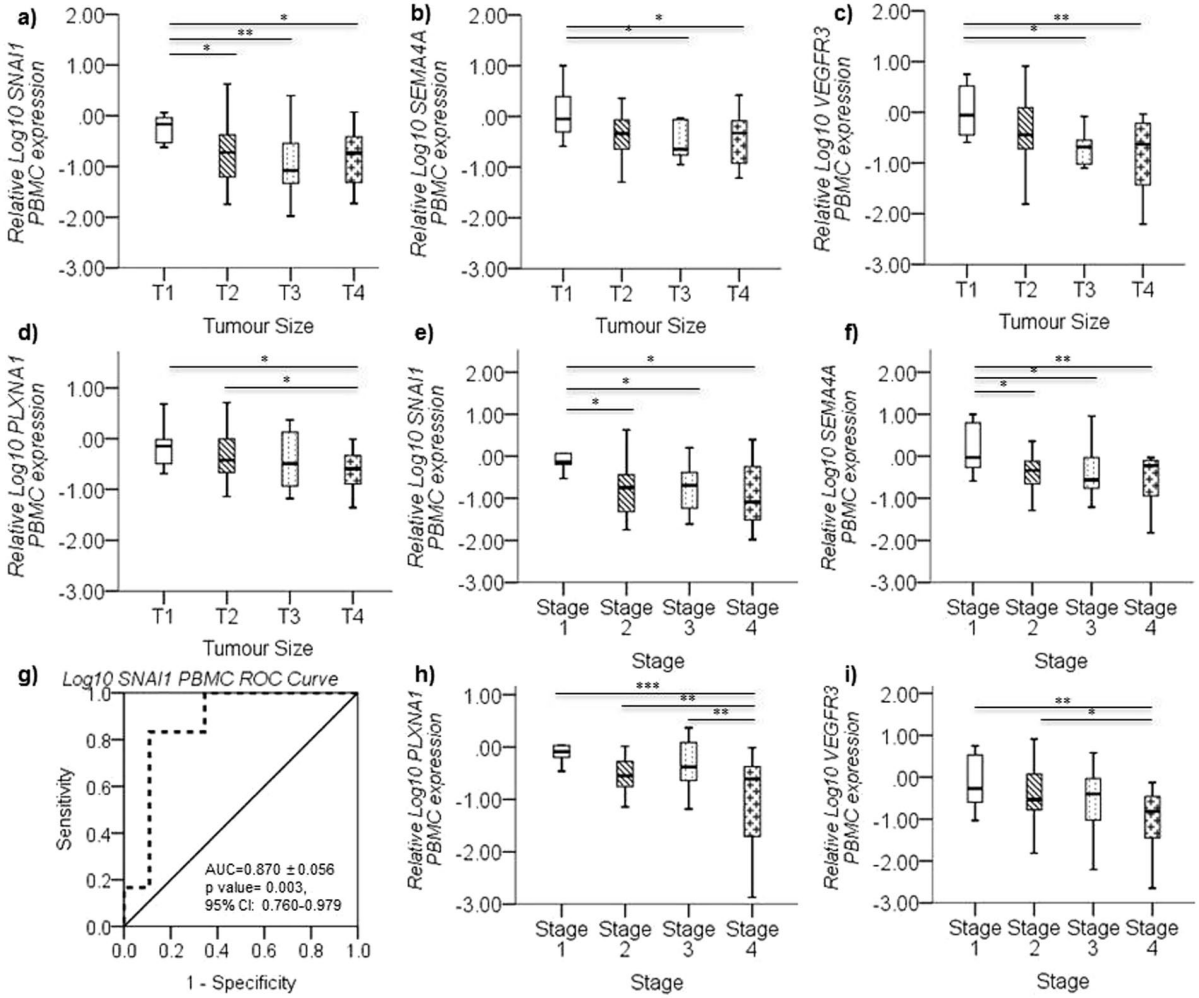
Decreased *SNAI1*, *SEMA4A*, *VEGFR3* and *PLXNA1* PBMC gene expression in patients with large tumor size and advanced disease stage. (**a**) The mean relative Log_10_ *SNAI1* expression ( ± SEM), as quantified by real time PCR, is significantly downregulated in PBMCs from breast cancer cases with tumor size ≥ 2 cm (T2, T3 and T4) compared to <2 cm (T1) (median value is represented by black line inside boxplot). Similarly, (**b**) Log_10_ *SEMA4A*, (**c**) Log_10_ *VEGFR3* and (**d**) Log_10_ *PLXNA1* mean relative gene expression decreases as the tumor size increases. (**e**) Mean relative Log_10_ *SNAI1* expression and (**f**) Log_10_ *SEMA4A* (±SEM) is significantly downregulated in advanced disease stages 2, 3 and 4 compared to Stage 1. (**g**) ROC analysis confirms that the expression of Log_10_ *SNAI1* in PBMCs differentiates between Stage 1 cases and all other advanced breast cancer stages (Stage 2–4) (AUC = 0.870 ± 0.056, p = 0.003, 95% CI: 0.760–0.979). Similarly, mean relative gene expression of (**h**) Log_10_ *PLXNA1* and (**i**) Log_10_ *VEGFR3* decreases in advancing disease with lowest expression in Stage 4 compared to the other stages. Target gene expression levels normalized against *GUSB* gene expression and scaled to expression levels in healthy controls. p < 0.05 considered to indicate statistical significance. *p < 0.05, **p < 0.01, ***p < 0.001, Multivariate ANOVA followed by Fisher’s LSD post hoc test.

On comparing PBMC expression profiles with patient disease characteristics, *SNAI1* expression in PBMCs indicated a significant decrease in cases harboring large tumor sizes T2 (*p* = *0.036*), T3 (*p* = *0.008*) and T4 (*p* = *0.041*) in comparison to patients presented with smaller tumor size T1 (Fig. 4a). Similarly, *SEMA4A* and *VEGFR3* expression in PBMCs was also downregulated significantly in cases with T3 (*p* = *0.020 & 0.021*) and T4 (*p* = *0.037 & 0.005*) tumor sizes compared to T1, however, tumors with size T2 did not show any significant difference in relation to both *SEMA4A* and *VEGFR3* expression in PBMCs compared to cases with T1 size tumors (Fig. 4b and c). Additionally, *PLXNA1* expression in PBMCs was significantly downregulated in cases with T4 tumor size compared to T1 (*p* = *0.030*) and T2 (*p* = *0.049*) (Fig. 4d).

### *SNAI1*, *SEMA4A*, *VEGFR3* and *PLXNA1* in PBMCs inversely relates with breast cancer stage

Similar to associations observed with tumor size, *SNAI1* and *SEMA4A* expression in PBMCs was significantly downregulated in patients with Stage 2 (*p* = *0.014 & 0.025*), Stage 3 (*p* = *0.020 & 0.013*) and Stage 4 (*p* = *0.036 & 0.009*) disease compared to Stage 1 patients as indicated by both multivariate (Fig. 4e and f) and univariate analysis (Table 2). ROC analysis further confirmed that *SNAI1* expression in PBMCs can differentiate Stage 1 disease from advanced stage disease (AUC = 0.870 ± 0.056, *p* = *0.003*, 95% CI: 0.760–0.979, log cut off = −0.1940, sensitivity = 83.3%, specificity = 89.1%) (Fig. 4g). Moreover, *PLXNA1* expression in PBMCs from Stage 4 cases was significantly downregulated compared to Stage 1 (*p* = *0.001*), Stage 2 (*p* = *0.010*) and Stage 3 (*p* = *0.005*) disease (Fig. 4h) (Table 2). *VEGFR3* expression was significantly downregulated in Stage 4 cases compared to Stage 1 (*p* = *0.009*) and Stage 2 (*p* = *0.040*) disease cases (Fig. 4i) (Table 2).

### *VEGFR3* and *PLXNA1* in PBMCs distinguish TNBC from other molecular subtypes

**Figure 5:**
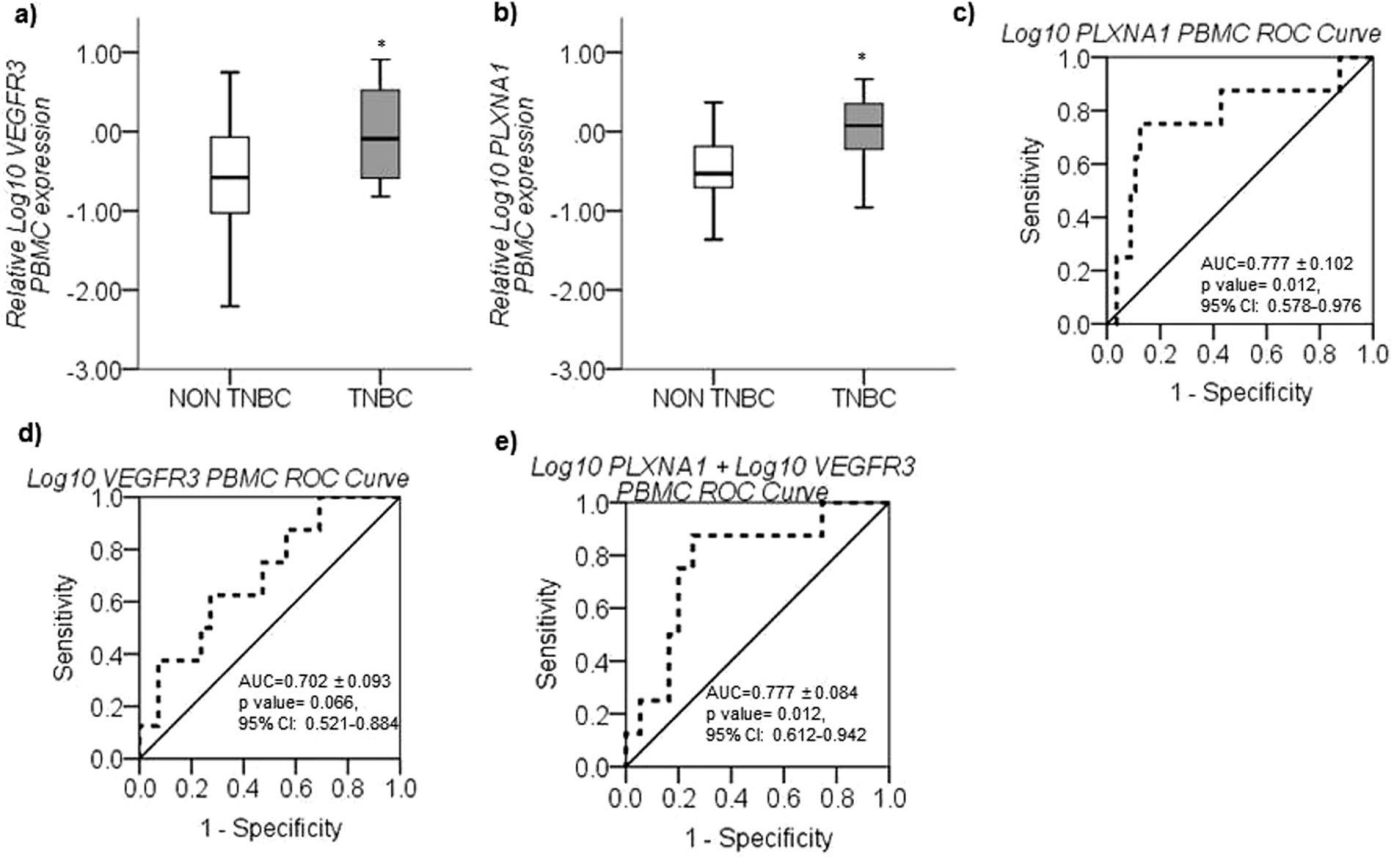
*VEGFR3 and PLXNA1* PBMC gene expression associated with TNBC. (**a**) Log_10_ *VEGFR3* and (**b**) Log_10_ *PLXNA1* mean relative gene expression (±SEM), quantified by real-time PCR, were significantly upregulated in PBMCs from TNBC compared to non-TNBC cases. (**c**) ROC analysis for TNBC cases against other breast cancer molecular subtypes based on PBMC expression of Log_10_ *PLXNA1* (AUC = 0.777 ± 0.102, p = 0.012), (**d**) Log_10_ *VEGFR3* (AUC = 0.702 ± 0.093, p = 0.066) and (**e**) a combination of Log_10_ *PLXNA1* + Log_10_ *VEGFR3* (AUC = 0.777 ± 0.084, p = 0.012). Combining Log_10_ *VEGFR3* and Log_10_ *PLXNA1* expression improved the specificity of the test to 74.5% from 57.1% while maintaining the high sensitivity (85.7%) to differentiate TNBC cases from other molecular subtypes. Target gene expression levels normalized against *GUSB* gene expression and scaled to expression levels in healthy controls. p < 0.05 considered to indicate statistical significance. *p < 0.05, Independent samples t-test (**a** and **b**).

On comparing PBMC expression profiles in TNBC against non-TNBC cases, *VEGFR3* and *PLXNA1* expression was observed to be significantly upregulated in TNBC (*p* = *0.044 & 0.017* respectively) (Fig. 5a and b). ROC analysis further indicated that *PLXNA1* but not *VEGFR3* expression in PBMCs could significantly distinguish TNBC patients from the other molecular subtypes (AUC = 0.777 ± 0.102, *p* = *0.012*, 95% CI: 0.578–0.976, log cut-off = −0.4318, Sensitivity = 85.7%, Specificity = 57.1%,) (Fig. 5c and d). However, combining *VEGFR3* and *PLXNA1* PBMC expression improved the specificity (74.5%) of the test, while maintaining the high sensitivity (85.7%, AUC = 0.777 ± 0.084, *p* = *0.012*, 95% CI: 0.612–0.942, log cut-off =−0.4599) (Fig. 5e).

### Early onset and older breast cancer patients show differential trends in plasma and PBMC expression profiles

**Figure 6:**
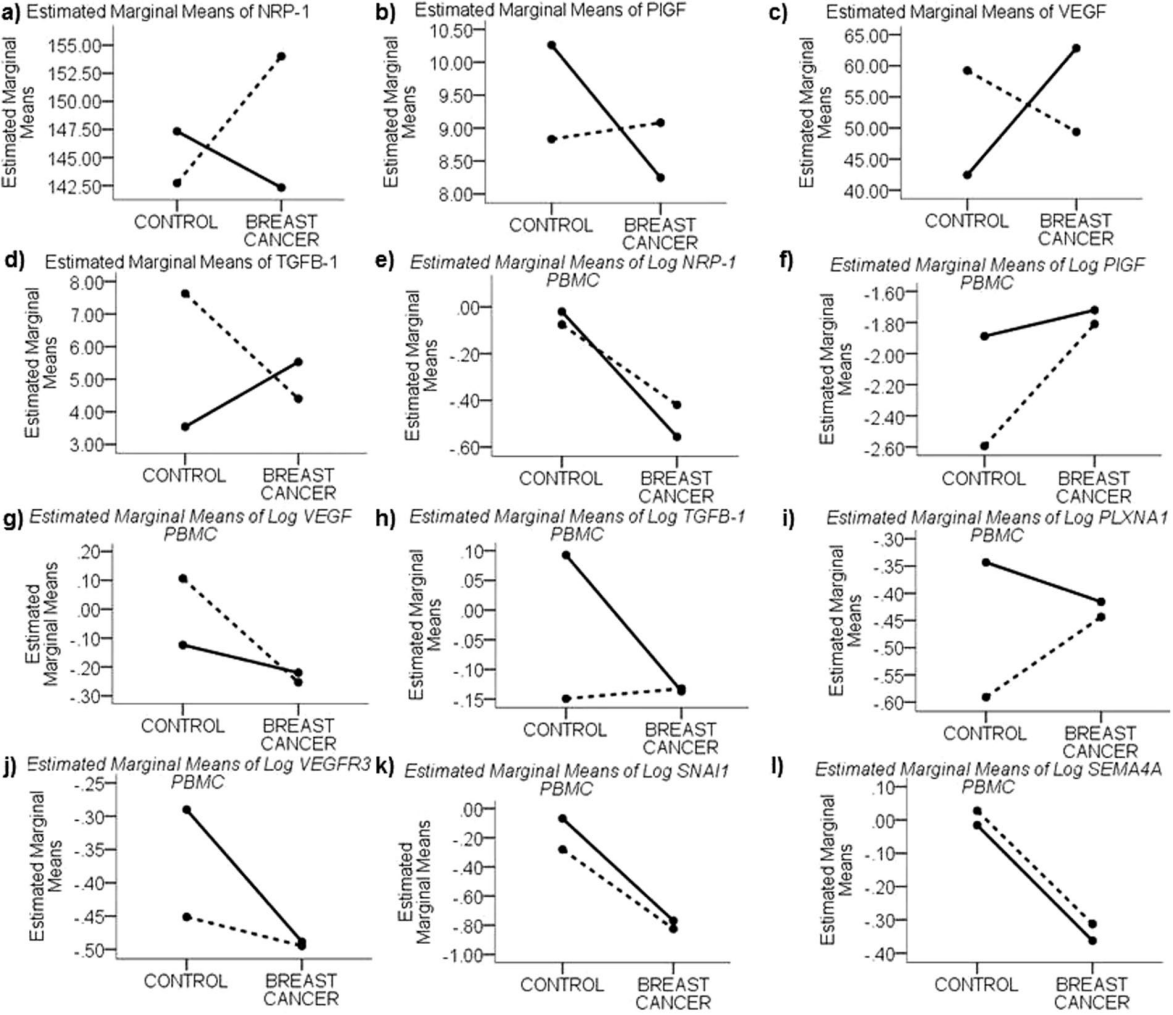
Age-dependent differential plasma proteins and PBMC expression profiles between breast cancer patients and healthy controls. Interaction plots of the estimated marginal means for soluble proteins (**a**) NRP-1, (**b**) PlGF, (**c**) VEGF and (**d**) TGFB-1; and estimated marginal means of relative log_10_ transformed gene expression in PBMCs of (**e**) *NRP-1*, (**f**) *PlGF*, (**g**) *VEGF*, (**h**) *TGFβ-1*, (**i**) *PLXNA1*, (**j**) *VEGFR3*, (**k**) *SNAI1* and (**l**)*SEMA4A* compared between early onset breast cancer patients (18–35 years) (solid black line) and older patients (>35 years) (dashed black line) vs age-matched healthy controls.

Univariate analysis to identify interactions between the age factor and the quantified plasma proteins and PBMC gene expression was conducted in both healthy controls and breast cancer patients. The estimated marginal means and analysis of variance between two main age groups i.e. early onset breast cancer patients (18–35 years) and older patients (>36 years) did not show any significant interactions however, it indicated interesting trends of differences between these age groups and their corresponding age-matched healthy controls (Fig. 6). Older breast cancer patients compared to early onset patients presented with higher plasma levels of both NRP-1 and PlGF (Fig. 6a and b). In addition, early onset patients displayed a decrease in plasma NRP-1 and PlGF in comparison to their age-matched controls whereas older patients displayed an increase. However, this trend was reversed for plasma VEGF and TGFβ1 since the early onset breast cancer patients had higher mean plasma VEGF and TGFβ1 compared to older patients (Fig. 6c and d). Moreover, compared to their respective age-matched controls, early onset cases displayed an increase in plasma VEGF and TGFβ1 whereas older patients displayed a decrease.

The expression of *NRP-1* in PBMCs decreased similarly in both early onset breast cancer cases and older cases compared to their respective age-matched controls (Fig. 6e). Contrary to observations in the plasma, a majority of the studied PBMC genes *PlGF* (Fig. 6f), *VEGF* (Fig. 6g), *TGFβ1* (Fig. 6h), *PLXNA1* (Fig. 6i) and *VEGFR3* (Fig. 6j), indicated similar expression in both early onset and older breast cancer patients, with controls showing higher variability between the two age groups.

Similar to *NRP-1* expression in PBMCs, some molecules appear to follow similar patterns of expression in PBMCs for instance, *SNAI1* (Fig. 6k) and *SEMA4A* (Fig. 6l) expression decreased in parallel in both age groups compared to their respective age-matched controls. *PlGF* (Fig. 6f) expression was upregulated in breast cancer patients irrespective of the patient age compared to healthy controls. However, *VEGF* (Fig. 6g) and *VEGFR3* (Fig. 6j) expression was downregulated in PBMCs from both early onset and older breast cancer patients in comparison to healthy controls. *TGFβ1* (Fig. 6h) and *PLXNA1* (Fig. 6i) expression was observed to be downregulated in early onset cases and upregulated in older patients compared to their age-matched controls.

## Discussion

The role of circulating biomarkers and differential gene expression profile in PBMCs in various cancers is widely recognized and investigated^5, 6, 22–25^. These disease-specific alterations stem from the ability of tumors to release signaling factors/cancer cells into the circulation and their ability to trigger or evade immunogenic responses. Considering the invasiveness, site-specificity and cost of true-cut biopsy based diagnosis; circulating biomarkers represent a lucrative and easily accessible compartment to identify disease-specific systemic changes in cancer patients. Based on the known functions of NRP-1 in tumor biology and immunosurveillance^26^, we focused in this study on quantifying NRP-1 and its related ligands and receptors in the plasma, breast tumor tissue and the corresponding gene expressions in PBMCs of breast cancer patients. We show that both plasma and tumor tissue NRP-1 is upregulated in patients with advanced diseased nodes and in metastatic breast cancer. Our result is consistent with a previous study which showed increased NRP-1 expression in breast cancer tumors tissues in patient cohorts with lymph node metastasis compared to patients with no involved nodes^27^. To the best of our knowledge, we are the first to show the dependence of upregulated plasma NRP-1 levels in breast cancer patients with advanced lymph node metastasis when more than 10 axillary lymph nodes or other nodes (infraclavicular, supraclavicular or internal mammary nodes) are involved (N3 disease). Similar to our results, Xin *et al*. have also reported increased plasma NRP-1 in metastatic breast cancer patients compared to healthy controls^13^, although we additionally show a significant upregulation in non-metastatic breast cancer patients compared to controls as well. NRP-1 expression has been correlated to metastasis in tumor tissue from prostate cancer patients only, not breast cancer^28^. These observations clearly highlight the role of both plasma and tumor tissue expression of NRP-1 in the EMT pathway, lymphatic metastasis and hence, advanced stage breast cancer.

We have further showed that the plasma level of PlGF, but not tumor tissue expression, is significantly upregulated in metastatic breast cancer patients compared to non-metastatic cases and healthy controls. A previous study found that PlGF is highly expressed in breast cancer tissue and in a large proportion of node positive breast cancer cases^29^. Moreover, Parr *et al*. illustrated that PlGF is upregulated in advanced stage breast cancer tissue, not plasma, and is significantly linked to metastasis and recurrence^30^. The level of plasma PlGF is also increased in colorectal cancers with large tumor size, diseased nodes^31^, and high risk of recurrence^32^. Similar to these findings, our study is the first to report upregulated plasma PlGF in metastatic breast cancer. Interestingly, we also report that both NRP-1 and PlGF are highly expressed in TNBC tumor tissue compared to luminal types of breast cancer, an observation that has been made previously for NRP-1 but not PlGF in primary breast tumors from the METABRIC study^33^.Thus, plasma and tumor tissue expression indicate that it is likely that NRP-1 and PlGF are both associated to function in concert to augment breast cancer metastasis to nodes and distant sites and hence, are indicators of poor prognosis. It is important to note that the plasma levels of NRP-1, PlGF, VEGF and TGF-β were not significantly different between the breast cancer patients and healthy controls in the studied cohort (Supplementary Fig. S2).

On the contrary, the decreased expression of *NRP-1* in addition to *SNAI1* and *SEMA4A* in PBMCs in the cancer cohort studied compared to healthy controls highlights their potential protective role against breast cancer. Further evidence is provided by our observation of decreasing *SNAI1* and *SEMA4A* expression with increased tumors size and overall disease stage. The NRP1/SEMA4A axis was identified by Delgoffe *et al*. to be critical in tumor infiltrating Treg cells, but not peripheral Treg cells, to maintain their stability and hence, evade anti-tumor immune responses^16^. Our observation of breast cancer-specific downregulation of both *NRP-1* and *SEMA4A* in PBMCs is in agreement with the reports of Delgoffe *et al*. that the interaction of these molecules on circulating PBMCs may not play a role in tumor immune evasion but rather has a protective characteristic. Hence, our observations of the NRP-1 profile in plasma, tissue and PBMCs in breast cancer highlight its multiple roles depending on the cell type. *SEMA4A* is constitutively expressed in plasmacytoid dendritic cells whereby it functions to activate the cytotoxic T cell response against external antigens^34^. Thus, the decreased expression of *SEMA4A* in PBMCs in our breast cancer cohort implies a malfunction in the adaptive immune response against tumor cells. The observed downregulation of *SNAI1* in PBMCs from advanced cases are similar to those found previously by Martin *et al*. in which they showed that breast cancer tissues with worse prognosis, high grade and node positivity exhibit downregulated SNAI1 expression^35^, which can be explained by the protective role of SNAI1 in normal breast tissues against malignancy^36^. Although the function of *SNAI1* in PBMCs has not yet been elucidated, Niu *et al*. reported that it is downregulated in PBMCs in rheumatoid arthritis (RA) and type 2 diabetes (T2D) patients and attributed its differential expression to immune response-related modulations^37^. Hence, it is likely that *SNAI1* plays a critical protective role in the circulating immune cells against diseases such as cancer, T2D and RA that are commonly characterized by an inflammatory component.

The expression of *VEGFR3* and *PLXNA1*, which are co-receptors of NRP-1, was observed to be significantly upregulated in PBMCs isolated from TNBC compared to non-TNBC cases. VEGFR3 is known to play a critical role in lymphangiogenesis^38^ and deregulations in the expression of the VEGF, semaphorin and plexin classes of ligands as well as receptors in TNBC tumor tissue was observed by Bender *et al*.^39^. However, the role of these molecules in PBMCs, especially in the context of TNBC, has not yet been elucidated. Interestingly, ROC analysis determined that the combined expression of *VEGFR3* and *PLXNA1* on PBMCs could differentiate TNBC cases from other breast cancer subtypes with a high sensitivity and specificity, providing evidence for a TNBC subtype-specific expression in PBMCs. This observation could be explained through two alternative hypotheses. It may indicate the potential presence of an interaction between VEGFR3 and Plexin A1 in PBMCs as co-receptors to enhance the binding of pro-tumorigenic ligands and stimulate VEGFR3 activity or tumor immune evasion and thus, negate the tumor suppressive nature of Plexin A1 signaling. It is interesting to note that the expression of both *VEGFR3* and *PLXNA1* in PBMCs was upregulated in smaller tumor sizes (<2 cm) and stage 1 disease in addition to being overexpressed in TNBC. Although this observation might appear contradictory, TNBC has been shown to be more immunogenic than other molecular subtypes i.e. it is most susceptible to lymphocytic immune infiltration due to activated adaptive immune responses hence improving overall and disease free survival^40, 41^. This property is common in early stage cancers wherein the anti-tumor immune responses are functional, albeit not necessarily effective enough^42^. Thus, as an alternative hypothesis, *VEGFR3* and *PLXNA1* expression on PBMCs could play a role as a biomarker to determine breast cancer cases susceptible to immunotherapy.

Considering the expression data obtained from the total PBMCs, we admit that the study would be stronger if we identified the white blood cell population responsible for the observed differential expression pattern of the studied molecules however; this was not possible due to the limitation in blood sample collection for this particular purpose. Previous studies showed that the T lymphocytes constitute up to 70% of the total PBMC population, with a predominant presence of the CD4+ T cells (25–60% of PBMCs), consisting of the Treg cells and T helper cells, compared to the CD8+ cytotoxic T cells (5–30% of PBMCs)^43^. Hence, it is probable that alterations in the CD4+ T cell compartment may drive changes in the total PBMC expression levels. On the other hand, there is a possibility of altered population distribution frequency of the PBMC subtypes in cancers, an aspect that also requires further investigation.

Although age was not detected to be a confounding factor in our study, we observed interesting trends in both plasma and PBMC gene expression profiles in early onset vs older breast cancer patients and also compared to healthy controls. These trends were particularly contrasting in the plasma profile since all the markers quantified in plasma i.e. NRP-1, VEGF, TGFβ1 and PlGF indicated opposing trends in early onset breast cancer vs older cases, with comparable variability in both healthy controls and breast cancer cases. This observation indicates the dynamic nature of the plasma compartment as a result of age-dependent physiological changes and is probably causal of the lack of significant differences in the plasma profile between healthy controls and breast cancer patients. Although, the impact of age was not captured statistically in terms of mathematical significance, these differences have important biological implications in personalized medicine. For example, the anti-VEGF agent bevacizumab, which was controversially withdrawn for metastatic breast cancer treatment by the FDA based on safety and efficacy issues^44^, might benefit more in early onset (<35 years) breast cancer patients since these patients clearly display upregulated circulating VEGF compared to healthy controls whereas patients >35 years have a similar or even slightly decreased plasma VEGF profile in cancer cases compared to controls. On the other hand, the mean difference between the younger and older females’ expression profiles in PBMCs appears much larger in healthy controls compared to women with breast cancer. This clearly implies that the role of the circulating/immune cells in cancer cases is transformed irrespective of the age of onset to enable tumor immune evasion.

Collectively, the observations from our study have several implications in the personalized diagnosis and treatment of breast cancer patients. Firstly, it highlights the potential to target circulating/tissue NRP-1 and PlGF that are associated with nodal and distant metastasis and advanced disease. Anti-NRP1 monoclonal antibodies are being tested in phase I clinical trials in advanced solid tumors^13, 45–47^ whereas an anti-PlGF molecule has recently shown promising results in *in vitro* and *in vivo* experiments^48, 49^. Our results support the clinical potential of these agents and highlight the need to investigate their benefit in combination specifically in advanced breast cancer. Secondly, this study implies the protective role of *NRP-1*, *SNAI1* and *SEMA4A* expression in PBMCs in healthy controls and early stage breast cancer patients. The role of NRP-1 thus appears to be specific to the compartments it is expressed in; acting as a risk factor in plasma and tumor tissue and as a protective factor in PBMCs. The molecular mechanism governing the ability of *SNAI1* and *SEMA4A* in PBMCs to safeguard the initiation or progression of breast cancer needs to be studied in further detail to determine methods to tap this defensive mechanism in treating cancer patients. Thirdly, our results emphasize on specific targets that can be developed for TNBC patients, who do not benefit from hormonal treatment, such as NRP-1, PlGF, VEGFR3 and Plexin A1. The role of Plexin A1 needs to be specifically studied in TNBC and in conjunction with VEGFR3 to determine if in this type of aggressive cancer the tumor suppressive activity of Plexin A1 is lost. Moreover, the ability of VEGFR3 to determine immunogenic tumors, a common characteristic in TNBC, also needs to be dissected. This will enable a biomarker-based categorization of patients that may benefit from immunotherapy. Fourthly, the age-related differences observed in the plasma profiles calls attention to the necessity to personalize targeted therapeutics not only dependent on the tumor subtype but also on the age. Further studies need to be conducted in a larger cohort size and in other ethnic populations to determine the consistency of our study results. Despite these limitations, our study highlights the prognostic value of circulating proteins, their expression in tumor tissue and gene expression in PBMCs and emphasizes on interesting drug candidates for poor prognosis breast cancer.

## Methods

### Patient cohort and sample collection

Patients diagnosed with histologically confirmed breast carcinoma at Sultan Qaboos University Hospital, who were chemo/surgery naïve, were included in the study cohort in the period between January 2015 and November 2016. Seventy female breast cancer patients and fifty age-matched healthy female volunteer controls with no known co-morbidities were enrolled in this study. The study was approved by the ethical committee at College of Medicine and Health Sciences, Sultan Qaboos University, (License #SQU.EU/162/14, MREC#1018). The committee approved, confirmed experiments, and informed signed consent was obtained from all participants and/or their legal guardians. All experiments were performed in accordance with the institutional and national guidelines. Prior to the commencement of any treatment 5 ml of blood was collected in ethylenediaminetetraacetic acid (EDTA) vacutainers. Patient’s personal information is very confidential and is not going to appear in anywhere. Patient clinical and pathological characteristics are listed in Table 1.

### Tumor size clinical assessment

Tumors were detected by the surgeon clinically and pathologically by a pathologist followed by the radiologist who performed ultrasound and mammogram. The size of the tumor was estimated based on the clinical investigation of the surgeon and confirmed on mammography and ultrasound using 3 dimensions including: anteroposterior (AP) × transverse (T) × length (L) such that, T1 (>2 cm) T2 (2–5 cm) T3 (>5 cm) and T4 (tumors breaching the chest wall/skin or inflammatory breast cancer).

### Serum soluble protein detection by ELISA

Patient blood collected in EDTA-coated vacutainers was subjected to density gradient centrifugation with Histopaque (Sigma Aldrich, UK) at 400 g with the break off for 30 min at room temperature. The separated plasma was frozen at −80 °C until further analysis. The concentration of soluble NRP-1, VEGF, TGFβ1 and PlGF were measured in the plasma samples using ELISA kits (R&D systems, USA) according to the manufacturer’s instructions. Standard curves (R^2^ > 0.98) using *E-coli* expressed recombinant human NRP-1/PlGF were run simultaneously in each experiment and were utilized to quantitatively determine the concentration of these molecules in the plasma (Supplementary Fig. S3a). Cell lysates with known NRP-1 expression from breast cancer cell lines (MDA-MB-231 > MCF-7 > BT-474) were used as controls to test the ELISA kit (Supplementary Fig. S3b).

### PBMC isolation and RNA extraction

Anti-coagulated blood was subjected to density gradient centrifugation with Histopaque (Sigma Aldrich, UK) for 30 min at 400 g (break off) at room temperature. The buffy coat layer containing peripheral blood mononuclear cells (PBMCs) was isolated, washed twice with cold PBS and pelleted at 250 g at 4 °C. RNA was extracted from the PBMCs using TRI reagent (Ambion, USA), phase separation with chloroform and overnight isopropanol precipitation. The RNA pellet was washed twice in 70% ethanol in DEPC water, dried completely and resuspended in DEPC-treated water (Ambion, USA). RNA was quantified using the NanoDrop^TM^ 2000c spectrophotometer (Thermo Scientific, USA) and RNA quality as indicated by the 260/280 was ensured to lie in the range of 1.8–2.0. 1 μg of extracted RNA was treated with DNase I (Ambion, Lithuania) for 15 min at room temperature and converted to cDNA using the high-capacity reverse transcription kit (Applied Biosystems, USA). Synthesized cDNA was diluted to a final concentration of 5 ng/ul in DEPC-treated water and stored at −80 °C until further analysis.

### Quantitative Real-Time PCR

Real-time PCR was conducted using the SoAdvanced mastermix (Biorad, USA). Primers were designed using the Primer Express software (Applied Biosystems, USA) and are listed in Supplementary Tables 1 and 2. 15 ng of cDNA was used per reaction. The CFX96 Real-time PCR system (Biorad, USA) was used under the following conditions: enzyme activation at 95  °C for 20 s and 40 cycles of Denaturing at 95 °C for 3 s and Annealing/Extension at 63.4  °C for 30 s. Specificity of PCR reactions was verified by melt curve analysis of each amplified product. Each real-time PCR reaction was performed in duplicate. A no template control (NTC) was performed for each primer pair tested in all experimental runs. Commercially available reference cDNA (Clontech, USA) was utilized as an inter-plate calibrator to identify technical variations between experimental runs. The generated Ct results were analyzed using the QBase data analysis software to generate relative expression values using the 2^−ΔΔCt^ method of calculation.

### GeNorm analysis

The most suitable reference genes utilized to normalize data for real-time PCR experiments in this study were identified from a set of 10 commonly used reference genes: *RPL13*, *B2M*, *ACTB*, *PUM1* ^50^, *GAPDH* ^51^, *HPRT1*, *TUBB2A* ^52^, *PPIA* ^53^, *GUSB* ^54^ and *PBGD* ^55^. The primers used are listed in Supplementary Table S1. The gene expression of the 10 test reference genes was analyzed in 14 representative patient cases and 17 healthy controls to determine their stability. The primers used to amplify NRP-1 and its related ligands and co-receptors related genes are listed in Supplementary Table S2. GeNorm analysis^56^ was conducted in the QBase data analysis software, which identifies the most stably expressed genes amongst the samples tested. The analysis indicated that the most stably expressed gene, with the least M value is *GUSB* and this gene was hereafter utilized to normalize expression data obtained from patient and control samples.

### Immunohistochemistry

Immunohistochemical analysis was conducted on 70 pathologically confirmed breast cancer tumor formalin-fixed paraffin-embedded tissue. Tissue sections of 3 μm were deparaffinized using xylene, rehydrated in graded ethanol (100%, 95% and 75%) and H_2_O. Antigen retrieval was performed using 1 mM Ethylenediaminetetraacetic acid (EDTA) (pH 9.0), in 95 °C water bath for 30–40 min. The activity of endogenous peroxidases was blocked with 2% hydrogen peroxide for 10 min. The slides were washed in PBS for 5 min followed by wash in 0.05% Triton-x100 and blocked in 5% goat serum (Dako, USA) and incubated with primary antibody (Anti-Neuropilin 1 antibody (ab81321) or anti-PLGF antibody (ab196666) (Abcam, UK) in a dilution of 1:200 at 4 °C overnight.

The slides were washed three times for 5 min in PBS and incubated with the EnVision™ + Dual Link System-HRP (Dako, USA) labeled secondary antibody for 30 min at room temperature. Another washing step in PBS was performed, followed by incubation with substrate chromogen solution (20 µl DAB + chromogen diluted in 1 ml DAB + substrate buffer; Dako) for 5 min. The sections were counterstained using hematoxylin solution for 2 min. The tissues were dehydrated using graded ethanol (75%, 95% and 100%) and xylene. Finally, the slides were mounted using DPX (Di-n-butyl phthalate in Xylene) (Sigma, USA). Tissues were visualized using (Nikon H600L) light microscope with digital camera (DS-Ri2). The imaging software used was (NIS Elements version 4.40). Immuno Reactive Scoring (IRS) was performed for the stained slides using the following formula: IRS = SI (staining intensity) x PP (% of positive cells). PP is defined as 0 if staining is negative, 1 if the positive cells are between 1–20%, 2 for staining between 21–50% and 3 for staining between 51–100%^57^. A negative primary antibody control slide was stained simultaneously to validate the specificity of the antibody staining (Supplementary Fig. S4a,b) and human placental tissue was stained with NRP-1 and PlGF as a positive control Supplementary Fig. S4c,d).

### Statistical analysis

The Shapiro-Wilk test for normality was conducted to determine the distribution of breast cancer patients in the cohort studied. While the distribution of the plasma protein dependent variables was confirmed to be normal, the PBMC gene expression data indicated non-normal distribution. Considering the heterogeneity in the population distribution shapes and unequal and limited number of cases amongst the subgroups studied, the PBMC gene expression dataset was log_10_ transformed to attain normality in the population distribution. Statistical analysis to identify differences in variables between healthy controls vs breast cancer patients was performed using the independent samples t-test, prior to which the Levene’s test for equality of variance was conducted to accept/reject the assumption of equal variances in the data. To determine the association between the plasma levels of the soluble proteins or their expression in the PBMCs or tumor tissue and the clinical/pathological characteristics of the breast tumors, a multivariate analysis of variance (MANOVA) was performed, followed by the Fisher’s least significant difference (LSD) post-hoc test. Results from MANOVA were further tested by univariate analysis to identify if the significant associations were due to individual effects or as a result of multiple interactions between molecules. The 70 chemo-naïve breast cancer patients were grouped according to the tumor molecular type (Luminal A, Luminal B, Luminal B-like, Her-2 type, Triple negative (TNBC)), proliferation index (Ki-67%), grade (Grade 1–3), tumor size (T 1–4), node status (N 0–3), metastasis status (M0, M1) and grouped TNM staging (Stage 1–4). Associations with a p < 0.05 were considered significant (95% Confidence Interval). Poorly represented subgroups (n < 3) were excluded from the analysis to avoid interpretation errors. The ability of PBMC expression of genes, individually or in combination, to distinguish between breast cancer subtypes/stages was determined through the Area Under the Curve (AUC) using Receiver Operating Characteristic (ROC) curve analysis. A range of cut-offs was tested for the ROC analysis to identify the most optimal sensitivity and specificity. Pearson’s correlation analysis was utilized with a two-tailed test to identify correlation between the tumor tissue NRP-1 and PlGF IRS values. The IBM SPSS software (version 22) was used for all statistical analysis and graphs preparation.

## Electronic supplementary material

Supplementary information
