# Supplementary material for: Neuropilin-1 Associated Molecules in the Blood Distinguish Poor Prognosis Breast Cancer: A Cross-Sectional Study
Source: Sci Rep. 2017 Jun 12;7:3301. doi: 10.1038/s41598-017-03280-0 (PMC5468252; doi:10.1038/s41598-017-03280-0)
Supplement: Supplementary file 1 — Supplementary information [file 41598_2017_3280_MOESM1_ESM.pdf]

Neuropilin-1 Associated Molecules in the Blood Distinguish Poor Prognosis Breast Cancer: A Cross-Sectional Study

Adviti Naik[1], Noura Al-Zeheimi[1], Charles Saki Bakheit[2], Marwa Al Riyami[3], Adil Al Ajarrah[4], Mansour S. Al Moundhri[5], Zamzam Al Habsi[4], Maysoon Basheer[1], Sirin A. Adham[1*]

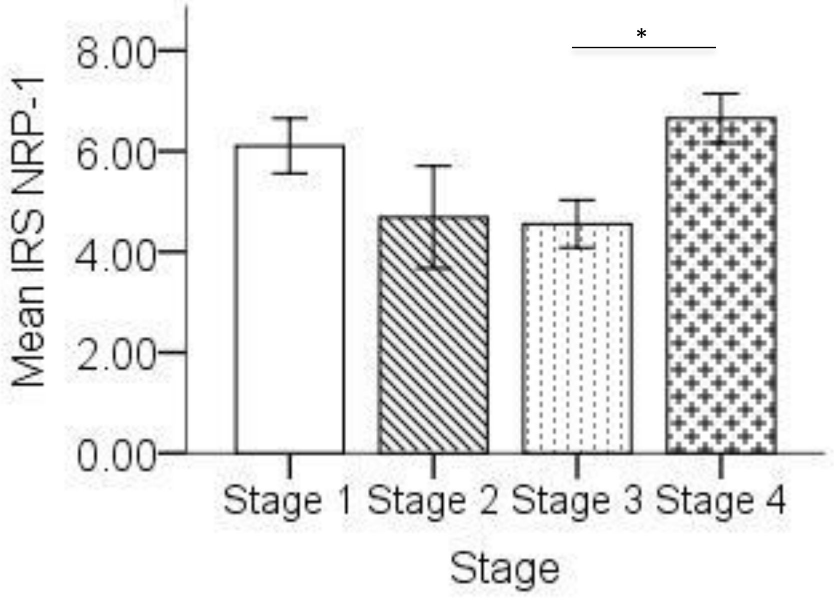

Supplementary Figure S1. Mean IRS score of NRP-1 in breast cancer stages. Graph represents calculated mean immuno-reactive score (IRS) of NRP-1 expression ± SEM in breast tumour tissue sub grouped according to overall disease stage. Stage 4 tumors were represented with higher levels of NRP-1 compared to stage 3 tumors *p= 0.012.*

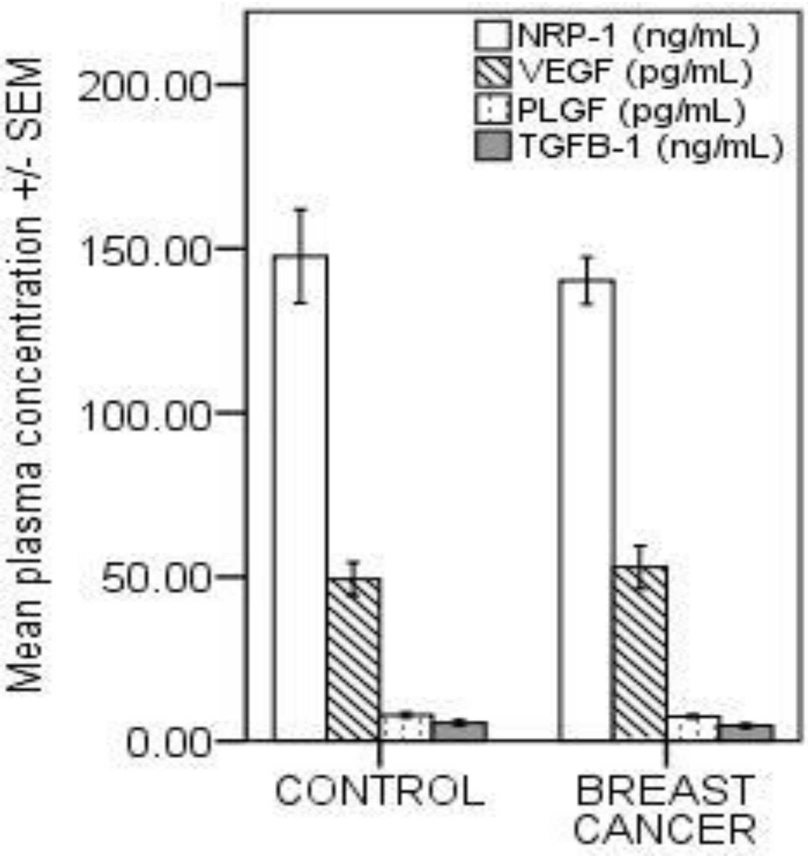

Supplementary Figure S2. Plasma NRP-1, PlGF, VEGF and TGFβ1 concentration in breast cancer and healthy controls. Graph represents a comparison of plasma concentration of NRP-1 (ng/mL), PlGF (pg/mL), VEGF (pg/mL) and TGFβ1 (ng/mL) (± SEM) between healthy controls (n=50) and breast cancer patients (n=70) as quantified by ELISA.

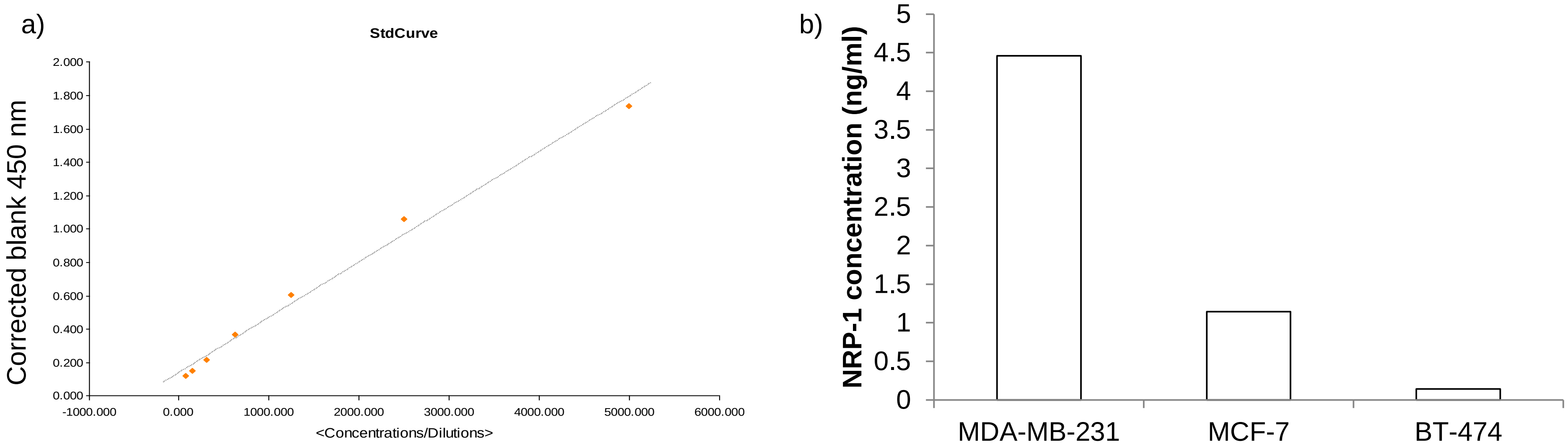

	Curve Formula	A	B	R2
StdCurve	Y=A*X+B	0.000331	0.138	0.99

Supplementary Figure S3. Validation of ELISA. a) Graph is a representative standard curve generated using recombinant NRP-1/PlGF to determine the specificity and dynamic range of the ELISA. b) NRP-1 concentration, as determined by ELISA, in cell lysates isolated from breast cancer cell lines (MDA-MB-231, MCF-7 and BT-474) with known relative NRP-1 expression confirm the specificity of the ELISA.

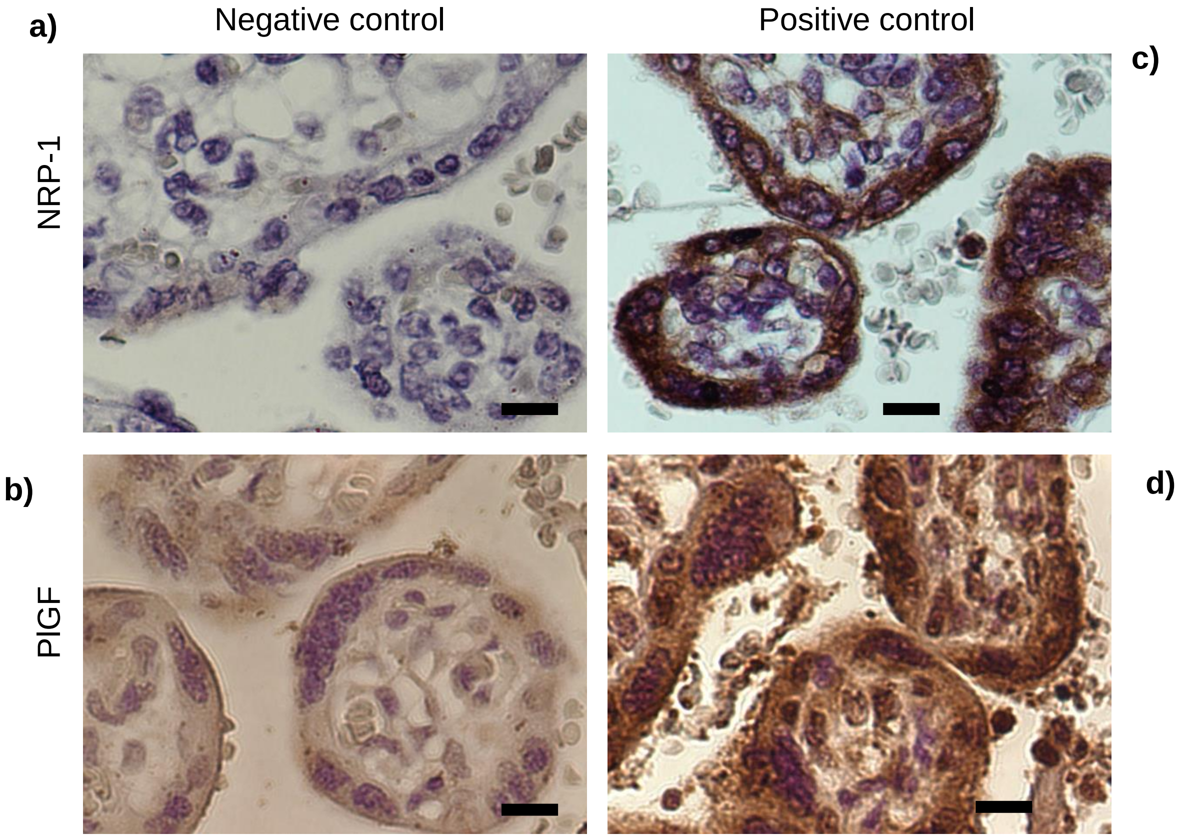

Supplementary Figure S4. Immunohistochemistry staining for normal human placental tissue as a positive **control for NRP-1.**Control placental tissue was simultaneously stained with breast tumour tissue to ensure the specificity of the staining. a) and b) are control negative staining for placental tissue were the primary antibody was not added to the sections serving as a back ground for staining. C) and d) is full staining with NRP-1 and PLGF respectively, staining show positive accumulation of both proteins in the placental tissue. Scale bar=50 μm

Supplementary Table 1. Candidate reference genes primer sequences

	Gene	Primer Sequences	Accession No.	T_m (^oC)
**1**	Ribosomal protein L13a (RPL13A), transcript variant 1	Fp: GAGGCCCCTACCACTTCC<br>Rp: AACACCTTGAGACGGTCCAG	NM_012423.3	65.7
**2**	Beta-2-microglobulin (B2M)	Fp: TAGGAGGGCTGGCAACTTAG<br>Rp: CTTATGCACGCTTAACTATCTTAACAA	NM_004048.2	55.4
**3**	Actin, beta (ACTB)	Fp: CCAACCGCGAGAAGATGA<br>Rp: CCAGAGGCGTACAGGGATAG	NM_001101.3	58.5
**4**	Pumilio RNA-binding family member 1 (PUM1), transcript variant 1	Fp: AGTGGGGGACTAGGCGTTAG<br>Rp: GTTTTCATCACTGTCTGCATCC	NM_001020658.1	46.3
**5**	Glyceraldehyde-3-phosphate dehydrogenase (GAPDH), transcript variant 1	Fp: CCTGGCCAAGGTCATCCATG<br>Rp: GGAAGGCCATGCCAGTGAGC	NM_002046.5	60.4
**6**	Hypoxanthine phosphoribosyltransferase 1 (HPRT1)	Fp: TTCTTTGCTGACCTGCTGG<br>Rp: TCCCCTGTTGACTGGTCAT	NM_000194.2	63.5
**7**	Tubulin, beta 2A class IIa (TUBB2A)	Fp: CTGGCACCATGGACTCTG<br>Rp: TCGGCTCCCTCTGTGTAG	NM_001069.2	64.4
**8**	Peptidylprolyl isomerase A (cyclophilin A) (PPIA), transcript variant 1	Fp: AGACAAGGTCCCAAAGAC<br>Rp: ACCACCCTGACACATAAA	NM_021130.4	61.4
**9**	Glucuronidase, beta (GUSB), transcript variant 1	Fp: GAAAATACGTGGTTGGAGAGCTCATT<br>Rp: CCGAGTGAAGATCCCCTTTTTA	NM_000181.3	59.4
**10**	Non-erythropoietic porphobilinogen deaminase(hydroxymethylbilane synthase (PBGD)	Fp: GGCTGCAACGGCGGAA<br>Rp: CCTGTGGTGGACATAGCAATGATT	X04808.1	56.6

Supplementary Table 2. Target genes primer sequences

	Gene	Primer Sequences	Accession No.
1	Placental growth factor (PlGF)	Fp: GGCCAATGTCACCATGCA<br>Rp: GAACGTCAGCTCCACGTAGGA	NM_002632
2	Semaphorin 3A (SEMA3A)	Fp: TTCAGCAATGGAGCTTTCCA<br>Rp: CAACCCCAGCCGTTGAAC	NM_006080.2<br>NM_002632
3	Semaphorin 4A (SEMA4A)	Fp: TTCTTTGAGAGGCTCCACACATC<br>Rp: CCGCCCACGTCATTCTTG	NM_022367.3
4	Plexin A1 (PLXNA1)	Fp: TCGATGGCAAGTCCGAGTACTT<br>Rp: CGAAGGGATCTTGAGCTGTGA	NM_032242.3
5	Vascular endothelial growth factor receptor 1 (VEGFR1)	Fp: AAAGGCACCCAGCACATCAT<br>Rp: TTCCCCCCTGCATTGGA	NM_002019.4
6	Vascular endothelial growth factor receptor 2 (VEGFR2)	Fp: CCTCCCCCGCATCACAT<br>Rp: GCTCGTTGGCGCACTCTT	NM_002253.2
7	Vascular endothelial growth factor receptor 3 (VEGFR3)	Fp: CAACGGCATCCAGCGATT<br>Rp: AAGGGATTTTCATGCACAATGA	NM_182925.4
8	E-cadherin	Fp: ACAGCCCCGCCTTATGATT<br>Rp: TGAAGGAAGCGGTTCCGA	NM_004360.4
9	N-cadherin	Fp: TGGGAATCCGACGAATGG<br>Rp: GCAGATCGGACCGGATACTG	NM_001792.4
10	Snail family transcriptional repressor 1 (SNAI1)	Fp: CCCCAATCGGAAGCCTAACT<br>Rp: GCTGGAAGGTAAACTCTGGATTAGA	NM_005985.3
11	Snail family transcriptional repressor 2 (SNAI2/SLUG)	Fp: CTGCGGCAAGGCGTTT<br>Rp: CGTGTGAGTTCTAATGTGTCCTTGA	NM_003068.4
12	Vimentin (VIM)	Fp: AATGACCGCTTCGCCAACT<br>Rp: ATCTTATTCTGCTGCTCCAGGAA	NM_003380.3
13	Neuropillin-1 (NRP-1)	Fp: CCAGGTCGAATCCGATCCT<br>Rp: CGCTGTCGGTGTAAAAAACCA	NM_003873.5
14	Zinc finger E-box binding homeobox 1 (ZEB1)	Fp: GCCAATAAGCAAACGATTCTG<br>Rp: TTTGGCTGGATCACTTTCAAG	NM_001128128.2
15	Vascular endothelial growth factor A (VEGF)	Fp: ATGACGAGGGCCTGGAGTGTG<br>Rp: CCTATGTGCTGGCCTTGGTGAG	NM_001171623.1
16	Transforming growth factor beta-1 (TGFB1)	Fp: AAGGACCTCGGCTGGAAGTG<br>Rp: CCGGGTTATGCTGGTTGTA	NM_000660.6
17	Hepatocyte growth factor (HGF)	Fp: CTCACACCCGCTGGGAGTAC<br>Rp: TCCTTGACCTTGGATGCATTC	NM_000601.5
